# Primary Measles Encephalitis

**DOI:** 10.21980/J80S75

**Published:** 2020-04-15

**Authors:** Milap Mehta, Maegan Reynolds, Jennifer Yee

**Affiliations:** * The Ohio State University, Department of Emergency Medicine, Columbus OH; ^ The Ohio State University, Nationwide Children’s Hospital, Columbus OH

## Abstract

**Audience:**

This scenario was developed to educate emergency medicine residents on the diagnosis and management of primary measles encephalitis.

**Introduction:**

Measles is a highly infectious ribonucleic acid (RNA) virus whose prevalence in the United States has continued to increase despite being declared eliminated in 2000,^1^ and larger outbreaks have been noted among those who elect not to vaccinate.^2^ The recommended live-attenuated measles, mumps, and rubella (MMR) vaccine schedule for pediatrics includes one routine dose at 12–15 months of age and a second dose between 4–6 years of age with at least 28 days in between dose administration.^1^–^2^ Measles-associated complications include otitis media, pneumonia, laryngotracheobronchitis, diarrhea, and corneal ulceration.^2^ Patients may also develop central nervous system complications, including primary measles encephalitis, acute post-infectious measles encephalomyelitis, measles inclusion body encephalitis, and subacute sclerosing panencephalitis. Primary measles encephalitis and measles inclusion body encephalitis involve an active ongoing measles infection.^3^ We will focus on primary measles encephalitis for this case scenario. One out of every 1000 measles patients will develop primary measles encephalitis,^1^ with onset typically occurring within seven days of the measles prodrome. Treatment is largely supportive. Mortality from primary measles encephalitis is 10%–15%, with an additional 25% developing permanent neurodevelopmental sequalae.^3^ It is critical to maintain a high index of suspicion for this diagnosis, to place the patient in airborne precautions to protect other immunocompromised individuals, and to transfer to a pediatric intensive care unit (PICU).

**Educational Objectives:**

At the conclusion of the simulation session, learners will be able to: 1) Obtain a relevant focused history, including immunization status, associated symptoms, sick contacts, and travel history. 2) Develop a differential for fever, rash, and altered mental status in a pediatric patient. 3) Discuss management of primary measles encephalitis, including empiric broad spectrum antibiotics and antiviral treatment. 4) Discuss appropriate disposition of the patient from pediatric emergency departments, community hospitals, and freestanding emergency departments, including appropriate time to call for transfer and the appropriate time to transfer this patient during emergency department (ED) workup. 5) Review types of isolation and indications for each.

**Educational Methods:**

This session was conducted using high-fidelity simulation, followed by a debriefing session and lecture on the diagnosis, differential diagnosis, and management of primary measles encephalitis. Debriefing methods may be left to the discretion of participants, but the authors have utilized advocacy-inquiry techniques. This scenario may also be run as an oral board case.

**Research Methods:**

Our residents are provided a survey at the completion of the debriefing session so they may rate different aspects of the simulation, as well as provide qualitative feedback on the scenario.

**Results:**

Feedback from the residents was overwhelmingly positive with an average score of 7 (consistently effective/very good or extremely effective/outstanding) across all categories. The subsequent debriefings allowed for multiple areas of discussion, including differential diagnoses of fever and rash, the clinical presentation of measles, empiric treatment of meningitis/encephalitis, types and indications of isolation, when to call for transfer to a pediatric center, and when a child is deemed stable enough for transfer.

**Discussion:**

This is a cost-effective method for reviewing primary measles encephalitis. There are multiple measles complications that may be reviewed via simulation, including pneumonia and dehydration from diarrhea. We encourage readers to utilize clinical photos of measles rashes, because this was difficult to capture via standard moulage techniques.

**Topics:**

Medical simulation, measles, primary measles encephalitis, encephalitis, infectious disease, emergency medicine, pediatric emergency medicine.

## USER GUIDE

[Table t1-jetem-5-2-s26]

**Table t1-jetem-5-2-s26:** 

List of Resources:	
Abstract	26
User Guide	28
Instructor Materials	30
Operator Materials	43
Debriefing and Evaluation Pearls	45
Simulation Assessment	50

**Learner Audience:**

Medical students, interns, junior residents, senior residents

**Time Required for Implementation:**

Instructor Preparation: 30 minutes

Time for case: 20 minutes

Time for debriefing: 40 minutes

**Recommended Number of Learners per Instructor:**

3–4

**Topics:**

Medical simulation, measles, primary measles encephalitis, encephalitis, infectious disease, emergency medicine, pediatric emergency medicine.

**Objectives:**

By the end of this simulation session, the learner will be able to:

1. Obtain a relevant focused history, including immunization status, associated symptoms, sick contacts, and travel history.
2. Develop a differential for fever, rash, and altered mental status in a pediatric patient.
3. Discuss management of primary measles encephalitis, including empiric broad spectrum antibiotics and antiviral treatment.
4. Discuss appropriate disposition of the patient from pediatric emergency departments, community hospitals, and freestanding emergency departments, including appropriate time to call for transfer and the appropriate time to transfer this patient during ED workup.
5. Review types of isolation and indications for each.

### Linked objectives and methods

Children with measles encephalitis require prompt recognition, treatment, and transfer to a pediatric care facility. In this case, providers will review how to diagnose measles encephalitis with an appropriately focused history and physical exam (objective 1) while still considering other differentials that may present similarly (objective 2). Participants should administer broad-spectrum antibiotics and antivirals to treat encephalitis and are expected to perform a lumbar puncture to obtain cerebrospinal fluid (CSF) for further testing (objective 3). Once the patient has received antibiotics and acyclovir and has been fluid-resuscitated, they should be transferred to a facility with a pediatric ICU (objective 4). Airborne precautions should be initiated as soon as participants consider measles as part of their differential (objective 5).

This simulation scenario allows learners to reinforce their primary measles encephalitis management skills in a psychologically-safe learning environment and then receive formative feedback on their performance.

### Recommended pre-reading for instructor

We recommend that instructors review literature regarding primary measles encephalitis, including epidemiology, presenting signs/symptoms, diagnosis, and management. The authors suggest starting with CDC resources, such as https://www.cdc.gov/measles/hcp/index.html. Other suggested readings include materials listed under the “References/suggestions for further reading” section below.

### Results and tips for successful implementation

This simulation was written to be performed as a high-fidelity simulation scenario, but also may be used as a mock oral board case.

The case was written for emergency medicine residents. We have conducted a similar primary measles encephalitis simulation case for approximately ten emergency medicine residents during the 2019–2020 academic year. While moulage was used to represent the fading rash, the authors feel that a picture of a clinical measles patient would better depict the patient’s skin lesions. Having N95 masks or powered air purifying respirators (PAPR) may also lend themselves to the fidelity of the case if airborne precautions are requested by participants.

Our simulation center’s feedback form is based on the Center of Medical Simulation’s Debriefing Assessment for Simulation in Healthcare (DASH) Student Version Short Form with the inclusion of required qualitative feedback if an element was scored less than a 6 or 7. This session had 5 feedback surveys returned. For all categories, the session received all 7 scores (consistently effective/very good or extremely effective/outstanding). Our form also includes an area for general feedback about the case at the end. The only qualitative comment stated “Great case with good learning points.”

## Supplementary Information



## Figures and Tables

**Table t2-jetem-5-2-s26:** 

#1	Complete blood count (CBC)
#2	Basic metabolic panel (BMP)
#3	Hepatic function panel
#4	Cerebrospinal fluid (CSF) studies
#5	Urinalysis
#6	Computed tomography (CT) head
#7	Chest X-ray (CXR)
#8	Measles rash
#9	Measles rash – close-up
#10	Koplik spots
#11	Measles: posterior oropharynx

**Table t3-jetem-5-2-s26:** 

**Vitals:**	Heart Rate (HR) 155	Blood Pressure (BP) 86/50	Respiratory Rate (RR) 24
	Temperature (T) 104.2°F	eOxygen Saturation (O_2_Sat) 98% on room air	
	Weight (Wt) 15 kg		

**Table t4-jetem-5-2-s26:** 

*Complete blood count (CBC)*	
White blood count (WBC)	15.4 x1000/mm^3^
Hemoglobin (Hgb)	14.0 g/dL
Hematocrit (HCT)	35.7%
Platelet (Plt)	580 x1000/mm^3^

**Table t5-jetem-5-2-s26:** 

*Basic metabolic panel (BMP)*	
Sodium	138 mEq/L
Chloride	99 mEq/L
Potassium	4.0 mEq/L
Bicarbonate (HCO_3_)	18 mEq/L
Blood Urea Nitrogen (BUN)	27 mg/dL
Creatinine (Cr)	1.1 mg/dL
Glucose	90 mg/dL
Calcium	8.0mg/dL

**Table:** 

*Liver Function Test (LFT)*	
Total bilirubin	1.2 mg/dL
Direct bilirubin	0.4 mg/dL
Albumin	3.0 g/dL
Alkaline Phosphate	100 U/L
Aspartate Aminotransferase (AST)	22 U/L
Alanine Aminotransferase (ALT)	40 U/L

**Table t7-jetem-5-2-s26:** 

*Cerebrospinal fluid (CSF) studies*	
Appearance	clear
Glucose	60 mg/dL
Protein	74 mg/dL
Red blood cells (RBC) (tube 4)	5–10 RBC/uL
Whitr blood cells (WBC)	400 WBC/uL
Gram stain	no organisms seen

**Table t8-jetem-5-2-s26:** 

*Urinalysis (UA)*	
Leukocyte esterase	negative
Nitrites	negative
Blood	none
Protein	none
Ketones	2+
Glucose	none
Color	dark yellow
White blood cells (WBC)	0–5 WBCs/high powered field (HPF)
Red blood cells (RBC)	0–5 RBCs/HPF
Squamous epithelial cells	0–5 cells/HPF
Specific gravity	1.015

**Figure f1-jetem-5-2-s26:**
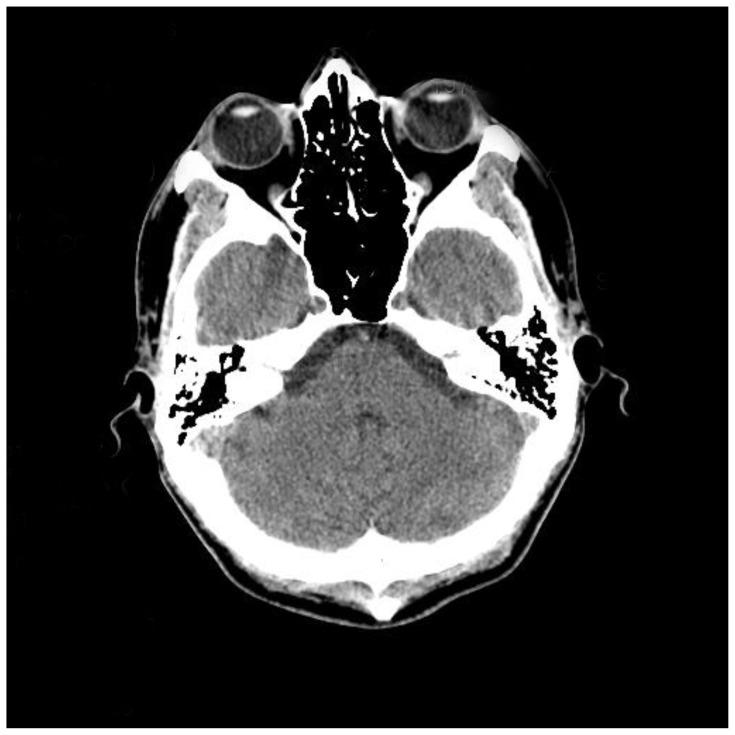


**Figure f2-jetem-5-2-s26:**
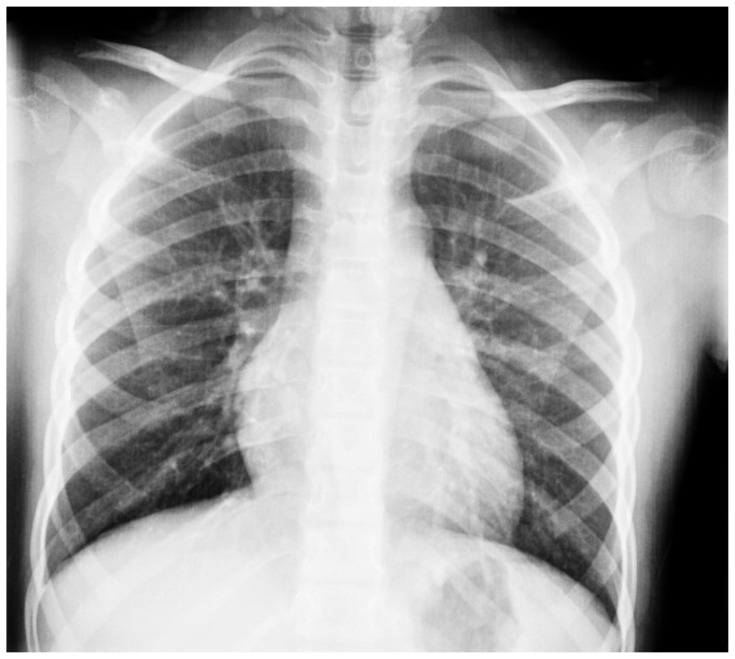


**Figure f3-jetem-5-2-s26:**
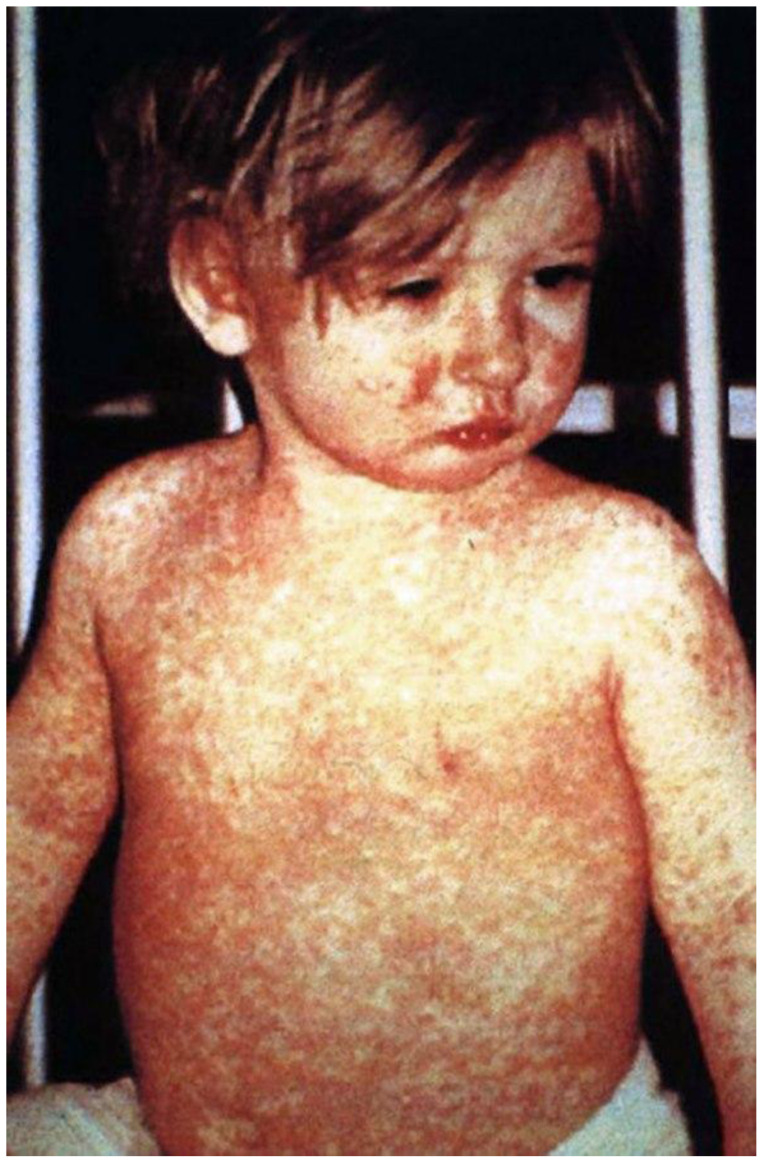


**Figure f4-jetem-5-2-s26:**
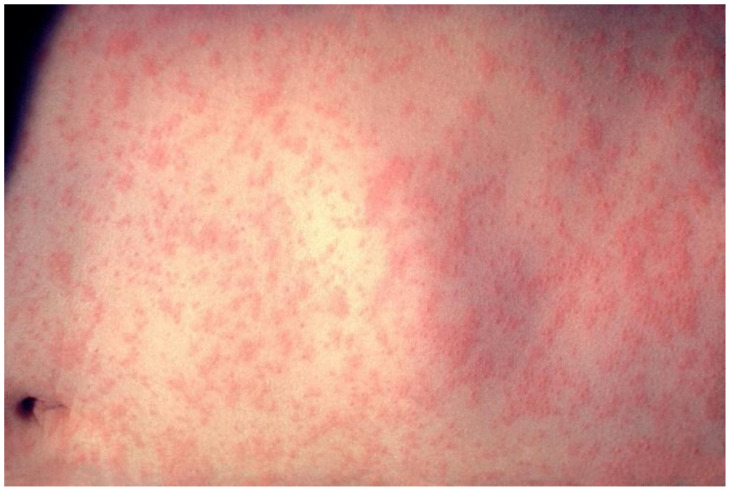


**Figure f5-jetem-5-2-s26:**
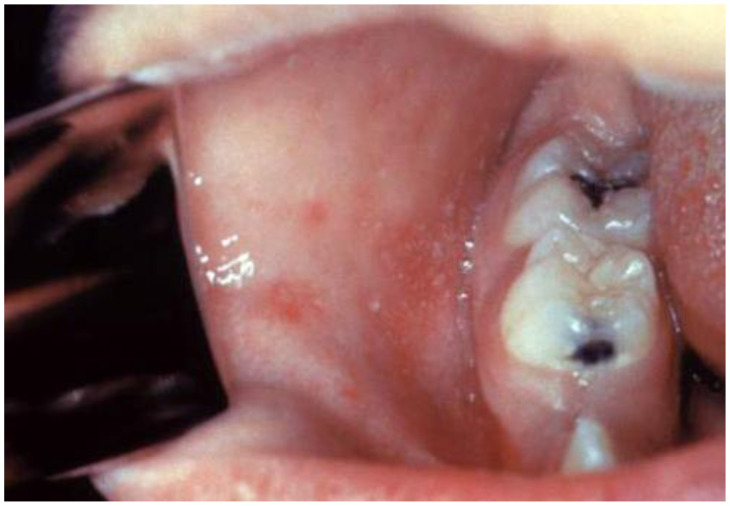


**Figure f6-jetem-5-2-s26:**
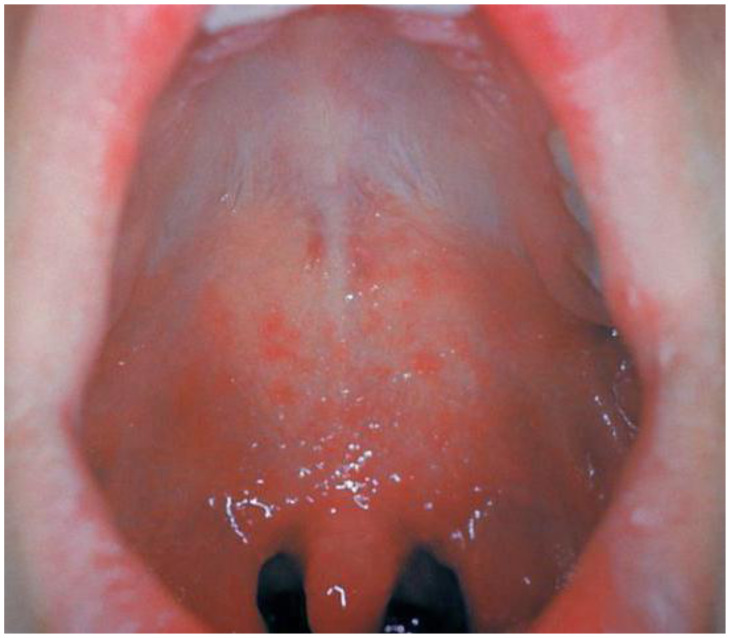


**Table t9-jetem-5-2-s26:** SIMULATION EVENTS TABLE:

Minute (state)	Participant action/ trigger	Patient status (simulator response) & operator prompts	Monitor display (vital signs)
0:00 (Baseline)	Patient moved into bed in the emergency department.	Participants should begin by placing the patient on a monitor, obtaining history from mom, and performing a physical exam.	A: T 104.2°F HR 155 BP 86/50 RR 24 O_2_sat 98% RA
4:00	IV placed, labs drawn. Participant should perform a thorough physical exam. Labs, UA, and CXR should be ordered.	If the team administers a 20 cc/kg bolus, tachycardia and hypotension will improve (B). If the team does not administer a 20 cc/kg bolus, tachycardia and hypotension will worsen (C).	
8:00	Team should suspect measles and place the patient in airborne precautions. Informed consent should be obtained from mom and LP be performed. Broad spectrum antibiotics should be given.	If team administers appropriate broad-spectrum antibiotics and antivirals, tachycardia and hypotension continue to improve (B). If team does not administer IV fluids, nursing may prompt, “I had a lot of trouble getting that IV, I think he’s pretty dry.” If team does not administer empiric antibiotics/antivirals, mom may prompt “how are you going to treat this?” If appropriate broad-spectrum antibiotics and antivirals are not given, vitals worsen to (E) vitals even if bolus previously given. Vitals will improve to (D) vitals once fluid bolus and antibiotics are given.	B: With 20 cc/kg bolus T 102.9°F HR 135 BP 100/70 RR 20 O_2_sat 98% RA C: Without 20 cc/kg bolus T 104.2°F HR 165 BP 70/40 RR 30 O_2_sat 98% RA
12:00 (B)	PICU should be contacted for transfer.	If the team has not yet verbalized their concern for primary measles encephalitis, the admitting team will ask what their differential is. They will mention that they have seen a picture of the patient’s rash in the chart and it looks concerning. Faculty may provide an even stronger prompt, including “it doesn’t look like a Neisseria meningitidis rash.” If a lumbar puncture isn’t performed, the admitting team should ask “what are your thoughts on an LP?” If IV fluid bolus and antibiotics/acyclovir not yet give, the PICU team will ask for continued resuscitation prior to transfer. If the patient is not in isolation, the PICU team will ask if they need isolation precautions for transfer.	D: With antibiotics (abx) and acyclovir T 102.9°F HR 120 BP 110/76 RR 20 O_2_sat 98% RA Without empiric abx and acyclovir T 104.2°F HR 155 BP 86/50 RR 24 O_2_sat 98% RA

**Table t10-jetem-5-2-s26:** 

**Critical Actions:** Place patient on monitor and obtain a full set of vitals, including temperature. Obtain point-of-care glucose. Obtain immunization status. Place the patient in airborne precautions. Obtain verbal consent from mom to perform lumbar puncture, including reviewing risks and benefits. Perform a lumbar puncture. Administer 20 cc/kg IV fluid bolus. Administer broad-spectrum antibiotics and antivirals appropriate for age-specific pathogens to treat encephalitis. Arrange for transport to a PICU.	0:00

**Table t11-jetem-5-2-s26:** 

	Milestone	Did not achieve level 1	Level 1	Level 2	Level 3
1	**Emergency Stabilization (PC1)**	□ Did not achieve Level 1	□ Recognizes abnormal vital signs	□ Recognizes an unstable patient, requiring intervention Performs primary assessment Discerns data to formulate a diagnostic impression/plan	□ Manages and prioritizes critical actions in a critically ill patient Reassesses after implementing a stabilizing intervention
2	**Performance of focused history and physical (PC2)**	□ Did not achieve Level 1	□ Performs a reliable, comprehensive history and physical exam	□ Performs and communicates a focused history and physical exam based on chief complaint and urgent issues	□ Prioritizes essential components of history and physical exam given dynamic circumstances
3	**Diagnostic studies (PC3)**	□ Did not achieve Level 1	□ Determines the necessity of diagnostic studies	□ Orders appropriate diagnostic studies. Performs appropriate bedside diagnostic studies/procedures	□ Prioritizes essential testing Interprets results of diagnostic studies Reviews risks, benefits, contraindications, and alternatives to a diagnostic study or procedure
4	**Diagnosis (PC4)**	□ Did not achieve Level 1	□ Considers a list of potential diagnoses	□ Considers an appropriate list of potential diagnosis May or may not make correct diagnosis	□ Makes the appropriate diagnosis Considers other potential diagnoses, avoiding premature closure
5	**Pharmacotherapy (PC5)**	□ Did not achieve Level 1	□ Asks patient for drug allergies	□ Selects an medication for therapeutic intervention, consider potential adverse effects	□ Selects the most appropriate medication and understands mechanism of action, effect, and potential side effects Considers and recognizes drug-drug interactions
6	**Observation and reassessment (PC6)**	□ Did not achieve Level 1	□ Reevaluates patient at least one time during case	□ Reevaluates patient after most therapeutic interventions	□ Consistently evaluates the effectiveness of therapies at appropriate intervals
7	**Disposition (PC7)**	□ Did not achieve Level 1	□ Appropriately selects whether to admit or discharge the patient	□ Appropriately selects whether to admit or discharge Involves the expertise of some of the appropriate specialists	□ Educates the patient appropriately about their disposition Assigns patient to an appropriate level of care (ICU/Tele/Floor) Involves expertise of all appropriate specialists
9	**General Approach to Procedures (PC9)**	□ Did not achieve Level 1	□ Identifies pertinent anatomy and physiology for a procedure Uses appropriate Universal Precautions	□ Obtains informed consent Knows indications, contraindications, anatomic landmarks, equipment, anesthetic and procedural technique, and potential complications for common ED procedures	□ Determines a back-up strategy if initial attempts are unsuccessful Correctly interprets results of diagnostic procedure
20	**Professional Values (PROF1)**	□ Did not achieve Level 1	□ Demonstrates caring, honest behavior	□ Exhibits compassion, respect, sensitivity and responsiveness	□ Develops alternative care plans when patients’ personal beliefs and decisions preclude standard care
22	**Patient centered communication (ICS1)**	□ Did not achieve level 1	□ Establishes rapport and demonstrates empathy to patient (and family) Listens effectively	□ Elicits patient’s reason for seeking health care	□ Manages patient expectations in a manner that minimizes potential for stress, conflict, and misunderstanding. Effectively communicates with vulnerable populations, (at risk patients and families)
23	**Team management (ICS2)**	□ Did not achieve level 1	□ Recognizes other members of the patient care team during case (nurse, techs)	□ Communicates pertinent information to other healthcare colleagues	□ Communicates a clear, succinct, and appropriate handoff with specialists and other colleagues Communicates effectively with ancillary staff

## INSTRUCTOR MATERIALS

**Case Title:** Primary Measles Encephalitis

**Case Description & Diagnosis (short synopsis):** Patient is an unimmunized 5-year-old healthy female who presents with fever and rash. Per mom, she began getting sick with a fever six days ago with subsequent development of runny nose, red watery eyes, and a cough. Four days ago, mom noticed a red spotty rash which began on her forehead and then spread downwards. Mom brings her in today because patient now won’t answer questions and keeps repeating that her head hurts. On exam, the patient is febrile and lethargic. Exam is notable for a brown maculopapular rash, which is confluent on the face and upper chest. Once measles is suspected, the patient should be placed in airborne precautions. Participants should obtain bloodwork, a chest-ray, urinalysis, and perform a lumbar puncture after informed consent is obtained from mom. Cerebrospinal fluid reveals an elevated protein level. Patient will become more tachycardic and hypotensive until fluid boluses and antibiotics are administered. Participants should administer broad-spectrum antibiotics and antivirals to cover for encephalitis and transfer to a hospital with pediatric ICU capabilities.

**Equipment or Props Needed:**

High fidelity pediatric simulation mannequin

Angiocaths for peripheral intravenous access = 18g, 20g, 22g

Cardiac monitor

Pulse oximetry

IV pole

Normal saline (1L x2)

Lactated Ringer’s (1L x2)

Broselow cart or bag, including Broselow tape

Simulated medications with labeling: ceftriaxone, vancomycin, acyclovir

Moulage for rash

**Confederates needed:**

Primary nurse and mother

**Stimulus Inventory:**

[Table t2-jetem-5-2-s26]

**Background and brief information:** Patient’s mother brings the 5-year-old patient to your small community hospital for fever and rash.

**Initial presentation:** The patient is a 5-year-old female with no past medical history other than unimmunized status who presents with 6 days of fever and one day of confusion. She has been holding her head and complaining of a headache today. Mom reports her rash first appeared four days ago. Mom denies any recent travel or sick contacts.

- Past medical history: none
- Past surgical history: none
- Medications: none
- Allergies: none
- Family history: noncontributory
- Vital signs:
  ○ HR – 155
  ○ Resp rate – 24
  ○ Temp – 104.2 F
  ○ BP – 86/50
  ○ Pulse ox – 98% on RA
- Weight: 15 kg

Assessment: Lying supine, drowsy but will open eyes to voice, intermittently moans and says “my head” and “mommy.” Diffuse maculopapular rash seen on exposed skin.

**How the scenario unfolds:** Patient is an unimmunized 5-year-old previously healthy female who presents with fever and rash. Per mom, she began getting sick with a fever 6 days ago with subsequent development of runny nose, red watery eyes, and a cough. Four days ago, mom noticed a red spotty rash which began on her forehead and then spread down. Mom brings her in today because patient now won’t answer questions and keeps repeating that her head hurts. On exam, the patient is febrile and lethargic. Exam is notable for a brown maculopapular rash, which is confluent on the face and upper chest. Once measles is suspected, the patient should be placed in airborne precautions. Participants should obtain bloodwork, a chest-ray, urinalysis, and perform a lumbar puncture after informed consent is obtained from mom. CSF reveals an elevated protein level. The patient will become more tachycardic and hypotensive unless IV fluid boluses and antibiotics are administered. Participants should administer broad-spectrum antibiotics and antivirals to cover for encephalitis and transfer to a hospital with pediatric ICU capabilities.

**Critical actions:**

1. Place patient on monitor and obtain a full set of vitals, including temperature
2. Obtain point-of-care glucose
3. Obtain immunization status
4. Place the patient in airborne precautions
5. Obtain verbal consent from mom to perform lumbar puncture, including reviewing risks and benefits
6. Perform a lumbar puncture
7. Administer 20 cc/kg IV fluid bolus
8. Administer broad-spectrum antibiotics and antivirals appropriate for age-specific pathogens to treat encephalitis

**Case Title:** Primary Measles Encephalitis

**Chief Complaint:** Patient is a 5-year-old female brought into your small community hospital by her mother from home for fever and rash.

[Table t3-jetem-5-2-s26]

**General Appearance:** Lying supine, drowsy but will open eyes to voice, intermittently moans and says “my head” and “mommy.” Diffuse maculopapular rash seen on exposed skin.

**Primary Survey:**

- **Airway:** intact
- **Breathing:** mildly tachypneic at rest
- **Circulation:** tachycardic rate and regular rhythm. 1+ symmetric pulses. Poor skin turgor. 3–4 second capillary refill.

**History:**

- **History of present illness:** Patient is a 5-year-old female who is brought into your small community hospital ED by her mother for fever. If mom is asked, patient has no significant past medical history, but has not received any vaccines due to maternal concern for autism. She has had a fever for six days and a rash for four days, which started on her forehead and then spread downwards. Highest fever at home was 104°F. She had a couple days of runny nose and a cough a few days ago. Her eyes were red, but this has slowly been improving. Mom noticed confusion today, as the patient cannot answer basic questions and just keeps saying “my head hurts.” Mom denies any recent travel or sick contacts.
- **Past medical history**: none
- **Past surgical history:** none
- **Medications:** none
- **Allergies:** None
- **Social history:** unimmunized due to mom’s concerns about autism
- **Family history:** noncontributory

**Assessment:** Lying supine, drowsy but will open eyes to voice, intermittently moans and says “my head” and “mommy.” Diffuse maculopapular rash seen on exposed skin.

**Secondary Survey/Physical Examination:**

- **General appearance:** Lying supine, drowsy but will open eyes to voice, intermittently moans and says “my head” and “mommy.” Diffuse maculopapular rash seen on exposed skin.
- **HEENT:**
  ○ **Head:** within normal limits other than rash seen on face (see skin)
  ○ **Eyes:** mild nonpurulent conjunctivitis
  ○ **Ears:** within normal limits
  ○ **Nose:** within normal limits
  ○ **Throat:** posterior oropharyngeal erythema with mild tonsillar hypertrophy. No tonsillar exudate. No stridor or drooling. No Koplik’s spots visualized.
- **Neck:** resists passive flexion. Trachea is midline. Mild cervical lymphadenopathy.
- **Heart:** regular and tachycardic, otherwise within normal limits
- **Lungs:** mildly tachypneic at rest, otherwise within normal limits
- **Abdominal/GI:** within normal limits
- **Genitourinary:** within normal limits
- **Rectal:** within normal limits
- **Extremities:** capillary refill 3–4 seconds, rash is present on extremities (see skin) but not present on palms/soles. No edema. Otherwise wnl.
- **Back:** rash is present on trunk and back (see skin)
- **Neuro:** Lethargic but awakens to voice. Confused and repetitive, saying “mommy” and “my head hurts.” Spontaneously moves all four extremities. No clonus. Cranial nerves intact. GCS 13 (E3V4M6).
- **Skin:** Brown erythematous maculopapular rash, confluent on the face and upper trunk. Rash is present everywhere other than mucosa, palms, and soles. No petechiae or purpura.
- **Lymph:** within normal limits
- **Psych:** within normal limits

**Results:**

[Table t4-jetem-5-2-s26]

[Table t5-jetem-5-2-s26]

[Table t5-jetem-5-2-s26]

[Table t7-jetem-5-2-s26]

[Table t8-jetem-5-2-s26]

*Head Computed Tomography*

Ciscel, A. Normal CT scan of the head; this slice shows the cerebellum, a small portion of each temporal lobe, the orbits, and the sinuses. In: Wikimedia Commons.

https://commons.wikimedia.org/wiki/File:Head_CT_scan.jpg. Published 12 August 2005.[Fig f1-jetem-5-2-s26]

*Chest Radiograph*

Author’s own image.[Fig f2-jetem-5-2-s26]

*Measles rash*

Center for Disease Control/Barbara Rice. Measles. In: Wikimedia Commons.

https://commons.wikimedia.org/wiki/File:RougeoleDP.jpg. Public domain.[Fig f3-jetem-5-2-s26]

*Measles rash – close-up*

Center for Disease Control/Dr. Heinz F. Eichenwald. Morbillivirus measles infection. In: Wikimedia Commons.

https://commons.wikimedia.org/wiki/File:Morbillivirus_measles_infection.jpg. Published in 1958. Public domain.[Fig f4-jetem-5-2-s26]

*Koplik spots*

Center for Disease Control. Koplik spots, measles 6111 lores. In: Wikimedia Commons.

https://commons.wikimedia.org/wiki/File:Koplik_spots,_measles_6111_lores.jpg. Published 1975. Public domain.[Fig f5-jetem-5-2-s26]

*Measles posterior oropharynx*

Bernard S. Enanthem in measles. In: Wikimedia Commons.

https://commons.wikimedia.org/wiki/File:Measles_enanthema.jpg. Published May 26, 2017. Public Domain.[Fig f6-jetem-5-2-s26]

## OPERATOR MATERIALS

[Table t9-jetem-5-2-s26]

### Diagnosis

Primary measles encephalitis

### Disposition

Transfer to a pediatric ICU

## DEBRIEFING AND EVALUATION PEARLS

### Primary Measles Encephalitis

#### Pearls

Any patient with altered mental status should be checked empirically for hypoglycemia early in their evaluation course.

#### Measles

- Measles is a highly infectious single-stranded enveloped RNA virus.^1^
- Previously eradicated in the United States; however, incidence has increased due to increasing rates of unvaccinated children in the Pacific Northwest, California, New York, Illinois, and Texas.^2^
- Prodrome: cough, coryza/rhinorrhea, nonpurulent conjunctivitis, and a fever that gradually increases and may exceed 39°C near the time of rash onset.
  ○ May see Koplik spots 1–2 days before/after onset of rash (blue-white spots on the buccal mucosa opposite of the molars), but these spots only last 12–72 hours.
- Rash: Often starts 3–5 days after prodrome. Rash lasts 5–6 days, and is a maculopapular rash that begins at the hairlines and then spreads down and out.
  ○ May become confluent as it spreads out.
  ○ Initially blanches, then may become non-blanchable.
  ○ Fades to brown, starting from the head and moving down and out.
  ○ Fever is present when the rash starts, which can help differentiate measles from other illnesses such as Kawasaki, scarlet fever, Fifth’s disease, or roseola.
  ○ Infectious until 4 days after rash onset.
- Most common complication: diarrhea.
- Most common cause of death: measles pneumonia (viral or superimposed bacterial).
- An initial history and physical exam should focus on the number of days of fever, the time of onset of fever in relation to rash onset, a thorough immunization history, sick contacts or travel to endemic areas. General history should also focus on hydration status, urine output, and other co-existing medical conditions.
- Physical examination should focus on overall appearance of the child as well as a full set of vital signs. Signs of dehydration (sunken eyes, dry mucosal membranes, diminished capillary refill, skin mottling, etc.) as well as characterization of the rash, presence or absence of Koplik spots, symmetry of breath sounds and adequacy of respirations should be noted.

#### Measles Encephalitis

- 0.1% of patients with measles develop acute primary encephalitis.^3^
- Often appears within a few days of the rash.
- Triad of fever, headache, altered mental status (uncomplicated measles cases should not have altered mental status).
- There is a wide differential diagnosis for fever and rash in a pediatric patient which includes, Neisseria meningitis, tick-borne or rickettsial illness, Stevens-Johnson’s syndrome/toxic epidermal necrolysis, staph scalded skin syndrome, mycoplasma, scarlet fever, chickenpox, rubella, viral exanthems, enterovirus, Kawasaki’s disease, etc.
  - Many pediatric rashes associated with fevers are related to self-limiting viruses. However, if the patient has signs of altered mental status relative to age, complications of the primary diagnosis must be considered. Complications include encephalitis, meningitis, electrolyte abnormalities such as hypoglycemia or hyponatremia, or sepsis with possible secondary complication of disseminated intravascular coagulation (DIC).
- Other rare encephalitides should be considered, including herpes encephalitis, anti-Nmethyl D-aspartate (NMDA) receptor encephalitis, and pediatric autoimmune neuropsychiatric disorders associated with streptococcal infections (PANDAS).^4^
  - Lumbar puncture with viral PCR (polymerase chain reaction) for the above pathogens in concert with consultation with a pediatric neurologist is recommended.
- Cerebral spinal fluid for measles encephalitis will show a marked lymphocytic pleocytosis and increased protein.
- Primary measles encephalitis treatment involves supportive care, but during initial workup, cover empirically with broad spectrum antibiotics/acyclovir for meningitis/encephalitis. Consider vancomycin, rocephin and acyclovir as initial empiric treatment depending on the age of the patient.
- Mortality from primary measles encephalitis is 5%–15%, and 25% have permanent neurodevelopmental sequelae.^3^

#### Subacute sclerosing panencephalitis

- Very rare degenerative CNS disease due to persistent infection with measles virus thought to be secondary to viral mutation. Occurs on average 7 years after measles infection. Universally fatal with death occurring within 3 years.^3^

#### Prevention

Vaccines: MMR is a live attenuated vaccine, often given at 12 months and 4–6 years of age.^5^

- One dose of MMR is 93% effective against measles, 78% effective against mumps, and 97% effective against rubella.
- Two doses of MMR are 97% effective against measles and 88% effective against mumps.

#### Treatment

- Severe measles cases among children, such as those who are hospitalized, should be treated with vitamin A.^1^ Vitamin A should be administered immediately on diagnosis and repeated the next day. The recommended age-specific daily doses are:
  ○ 50,000 IU for infants younger than 6 months of age
  ○ 100,000 IU for infants 6–11 months of age
  ○ 200,000 IU for children 12 months of age and older
- Suspected measles? Place in **airborne** precautions!
  ○ Negative air pressure isolation room
  ○ Well-fitted respirator mask (N95 or PAPR)

#### Quarantine and Reporting

- Consider immune status of the individual being quarantined, presumptive evidence of immunity, whether the person is at high risk or not, and transmission settings.
- Consider immune status of healthcare providers and appropriate isolation and post-exposure prophylaxis, if indicated.
- Report any suspected case to the local health department who can assist in quarantine, notifying others possibly exposed, confirming diagnosis, and coordinating post-exposure prophylaxis.
- Diagnosis can be confirmed through a PCR throat swab in conjunction with serum IgM measles antibodies.
- MMR vaccine within 72 hours of measles exposure or immunoglobulin within six days of exposure may provide some protection or modify clinical course of disease among susceptible persons.^5^
  ○ Do not administer MMR vaccine and immunoglobulin simultaneously, since this will invalidate the vaccine.

#### Isolation^6^

- Contact precautions: prevent transmission of infectious agents spread by direct or indirect contact with the patient or their environment.
  ○ At least 3 feet of spatial separation between beds. Healthcare personnel should wear a gown and gloves for all interactions that may involve contact with the patient or potentially contaminated areas in the patient’s environment.
  ○ Organisms calling for this type of isolation: enterohemorrhagic *Escherichia coli* O157:H7, *Shigella spp*, hepatitis A virus, noroviruses, rotavirus, *Clostridium difficile, Staphylococcus aureus (methicillin-sensitive or -resistant), group A streptococcus*, Ebola, respiratory syncytial virus (RSV), parainfluenza virus, adenovirus, influenza, *mycobacterium tuberculosis*, severe acute respiratory syndrome virus (SARS), avian influenza, *Streptococcal pneumoniae*, varicellazoster, *herpes simplex*, variola (smallpox), Ebola.
- Droplet precautions: prevent transmission of pathogens spread through close respiratory or mucus membrane contact with respiratory secretions, but pathogens do not remain infectious over long distances in a healthcare facility.
  ○ Separate patient beds at least 3 feet and draw the curtain between beds. Healthcare personnel should wear a mask for close contact with infectious patients.
  ○ Organisms calling for this type of isolation: *Neisseria meningitidis* (first 24 hours of antimicrobial therapy), Ebola, respiratory syncytial virus (RSV), rhinovirus, parainfluenza virus, adenovirus, influenza, *Bordetella pertussis*, group A streptococcus.
- Airborne precautions: prevent transmission of infectious agents that may remain infectious over long distances when suspended in the air.
  ○ Place in an airborne infection isolation room that has special air handling and ventilation capacity. Mask the patient and provide N95 filtering facepiece respiratory mask or higher-level respirators (PAPR) for healthcare personnel.
  ○ Organisms calling for this type of isolation: Rubeola virus (measles), varicella virus (chickenpox), *mycobacterium tuberculosis*, severe acute respiratory syndrome virus (SARS), avian influenza, variola (smallpox).

#### Disposition

- All suspected cases of measles and measles encephalitis should be admitted to the nearest pediatric hospital with capabilities for infectious disease consultation and airborne isolation. Admission to a PICU should be considered for cases of measles encephalitis given the risk of increased morbidity and mortality.
- Remember: if you are not at a primary pediatric ED and you have a sick pediatric patient, call for transfer early (ie, as soon as child is recognized to be “sick” and admission recommended) and use your Broselow cart/bag!
- Before transferring, prioritize treatments that have an effect on decreasing morbidity, such as fluid resuscitation and antibiotic administration.

#### Other debriefing points

Closed-loop communication amongst team: was it used? Why or why not? Were there any implications of this during case execution?

**Wrap Up:** *Brief wrap up lecture (optional), references and/or suggestions for further reading. Please also include any other optional associated content here (worksheets for observing learners, etc).*

## SIMULATION ASSESSMENT

*Primary Measles Encephalitis*

Learner: _________________________________________

***Assessment Timeline***

This timeline is to help observers assess their learners. It allows observer to make notes on when learners performed various tasks, which can help guide debriefing discussion.

[Table t10-jetem-5-2-s26]

**Critical Actions:**

□ Place patient on monitor and obtain a full set of vitals, including temperature.
□ Obtain point-of-care glucose.
□ Obtain immunization status.
□ Place the patient in airborne precautions.
□ Obtain verbal consent from mom to perform lumbar puncture, including reviewing risks and benefits.
□ Perform a lumbar puncture.
□ Administer 20 cc/kg IV fluid bolus.
□ Administer broad-spectrum antibiotics and antivirals appropriate for age-specific pathogens to treat encephalitis.
□ Arrange for transport to a PICU.

**Summative and formative comments:**

**Milestones assessment:**

[Table t11-jetem-5-2-s26]
