# Supplementary material for: Primary Measles Encephalitis
Source: J Educ Teach Emerg Med. 2020 Apr 15;5(2):S26–54. doi: 10.21980/J80S75 (PMC10332560; doi:10.21980/J80S75)
Supplement: Supplementary file 1 [file jetem-5-2-s26-supp1.pptx]

## Slide 1
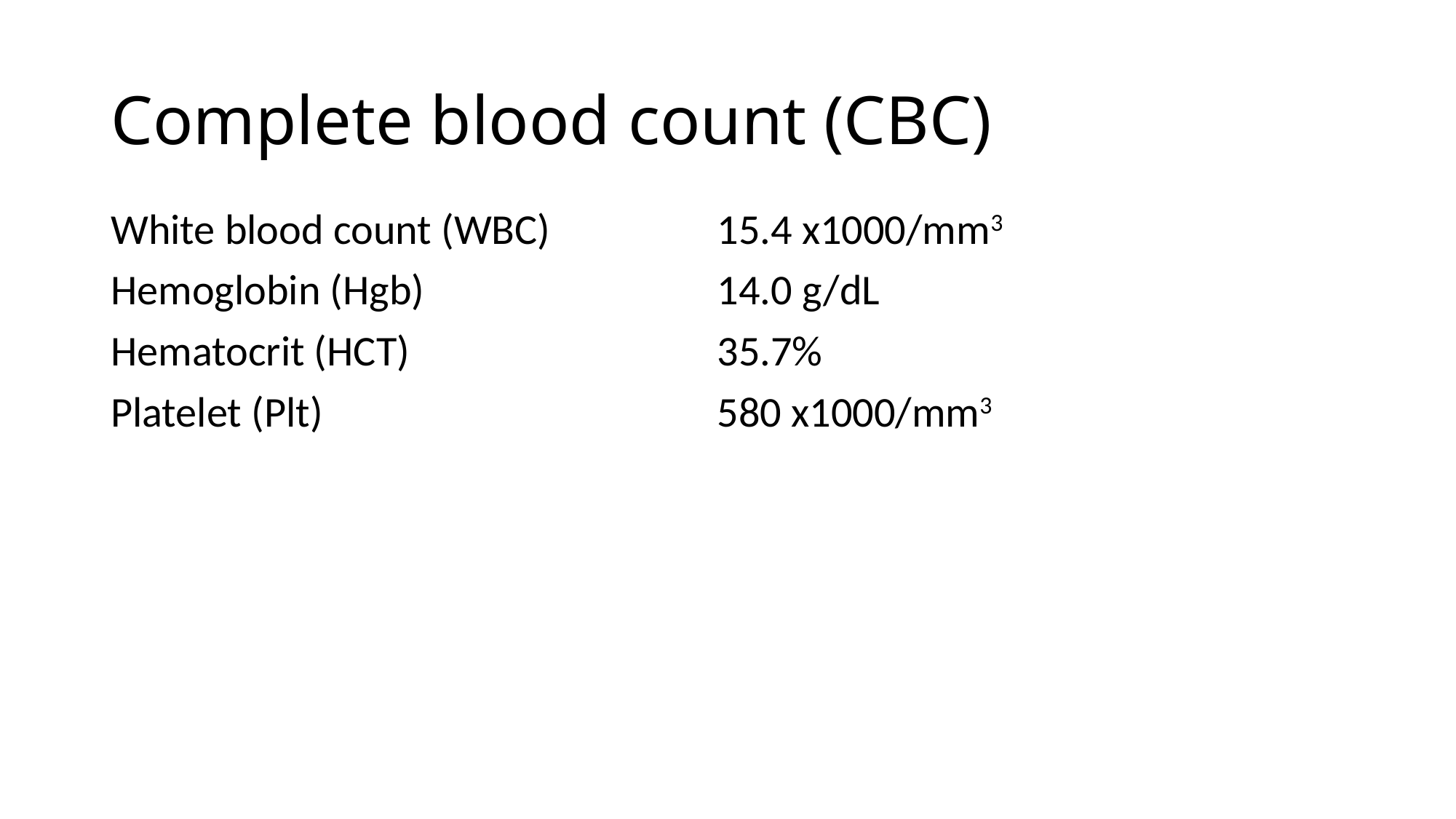

# Complete blood count (CBC)
White blood count (WBC)
Hemoglobin (Hgb)
Hematocrit (HCT)
Platelet (Plt)
15.4 x1000/mm3
14.0 g/dL
35.7%
580 x1000/mm3

## Slide 2
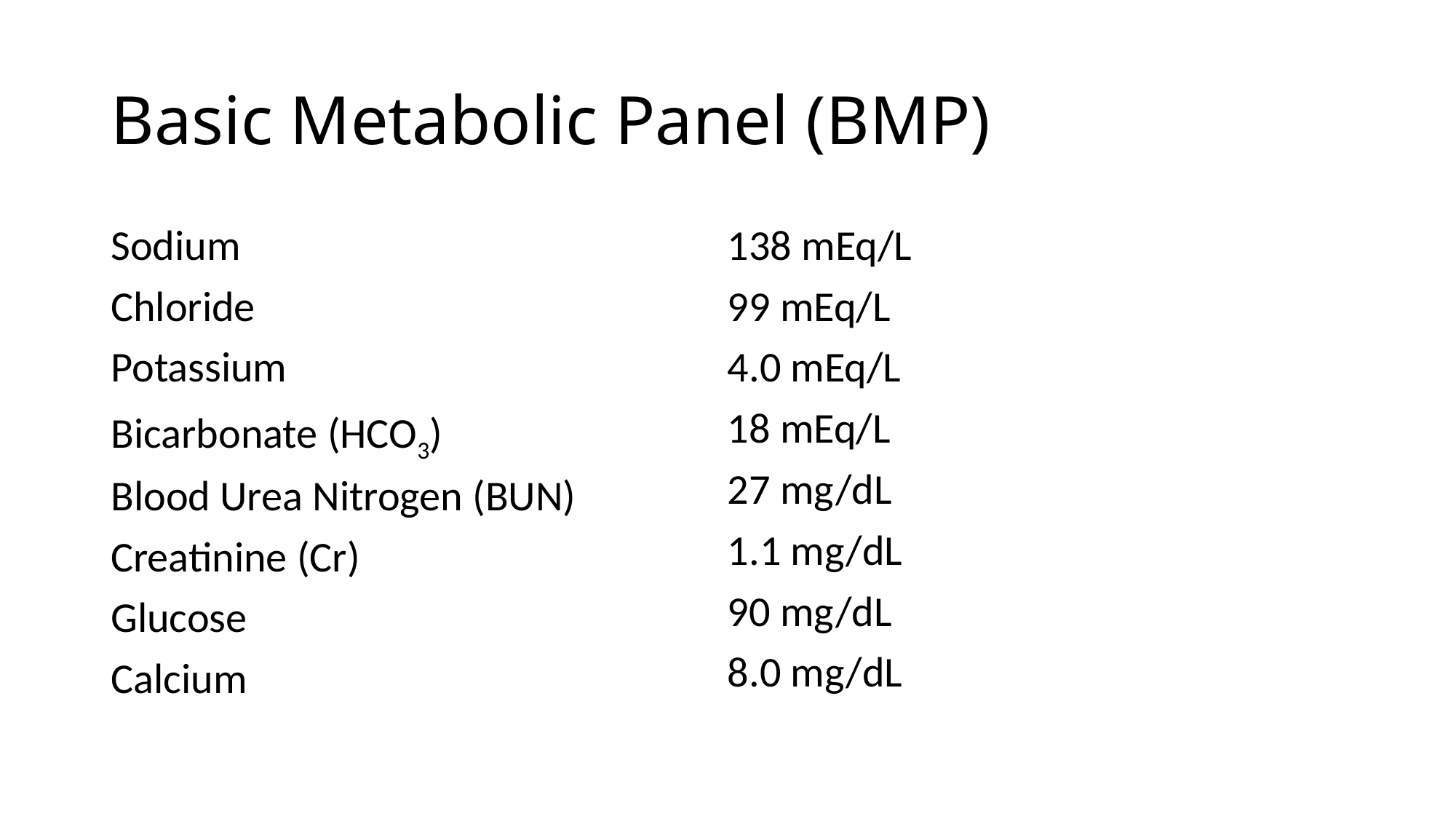

# Basic Metabolic Panel (BMP)
Sodium
Chloride
Potassium
Bicarbonate (HCO3)
Blood Urea Nitrogen (BUN)
Creatinine (Cr)
Glucose
Calcium
138 mEq/L
99 mEq/L
4.0 mEq/L
18 mEq/L
27 mg/dL
1.1 mg/dL
90 mg/dL
8.0 mg/dL

## Slide 3
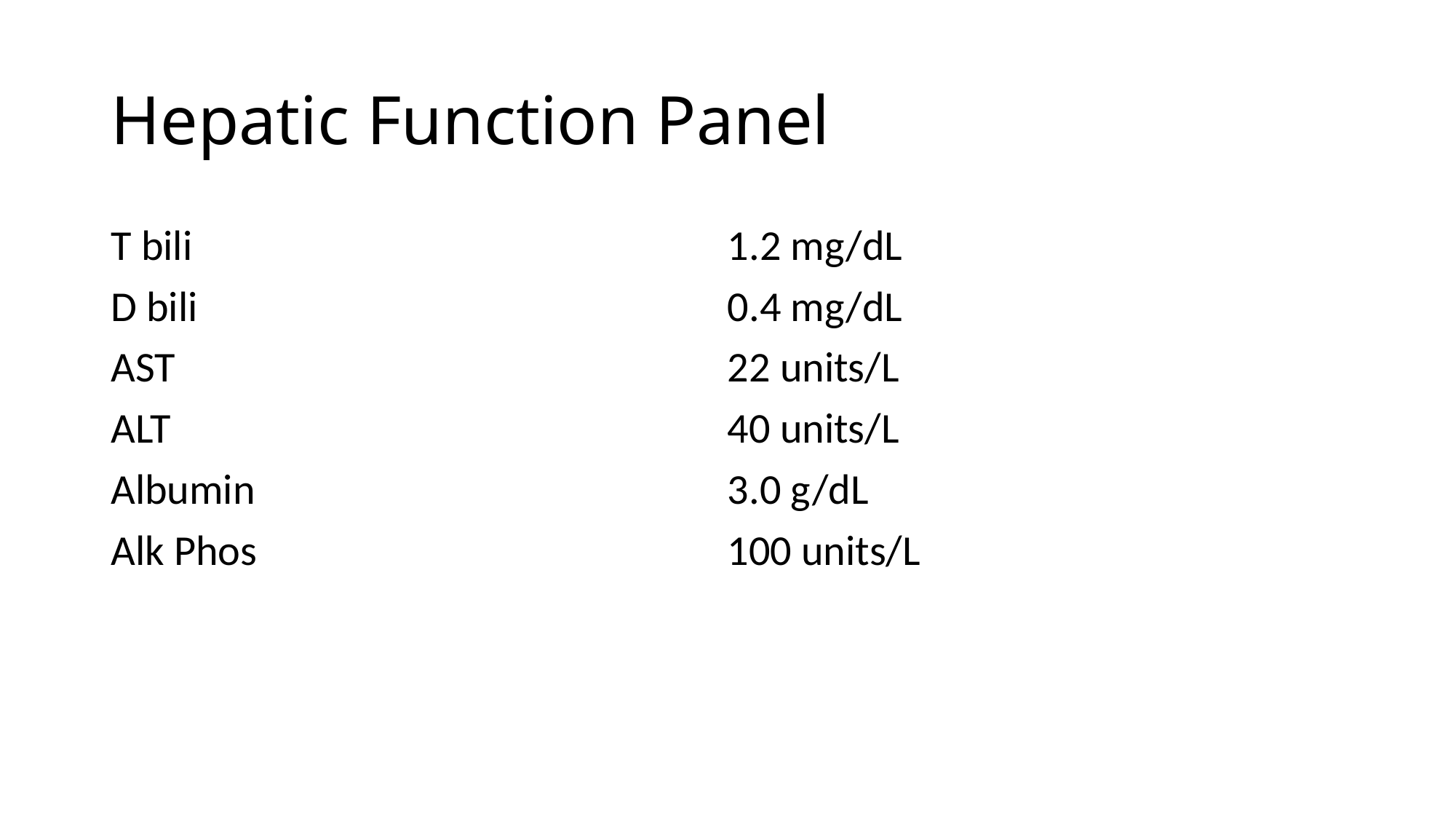

# Hepatic Function Panel
T bili
D bili
AST
ALT
Albumin
Alk Phos
1.2 mg/dL
0.4 mg/dL
22 units/L
40 units/L
3.0 g/dL
100 units/L

## Slide 4
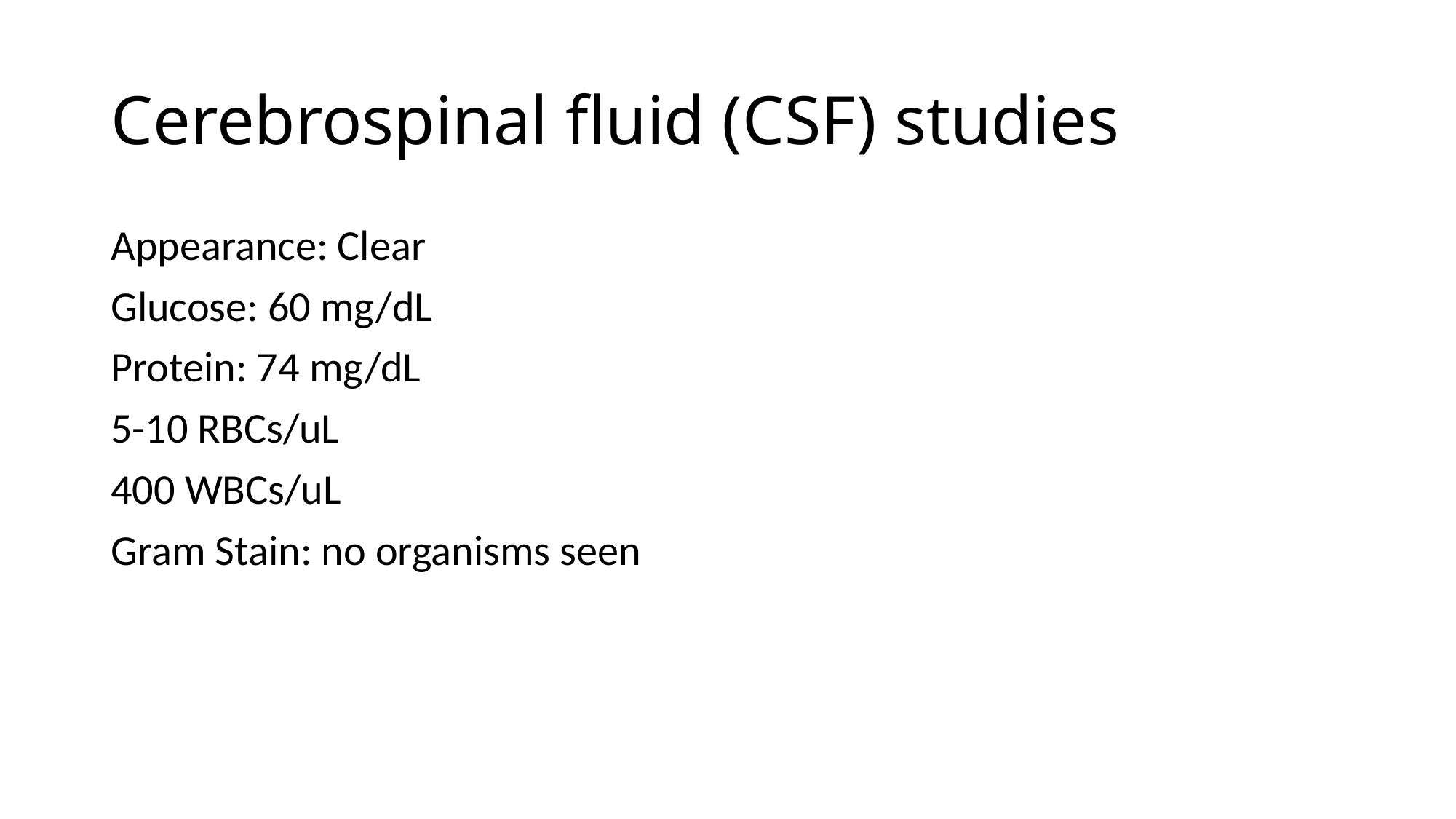

# Cerebrospinal fluid (CSF) studies
Appearance: Clear
Glucose: 60 mg/dL
Protein: 74 mg/dL
5-10 RBCs/uL
400 WBCs/uL
Gram Stain: no organisms seen

## Slide 5
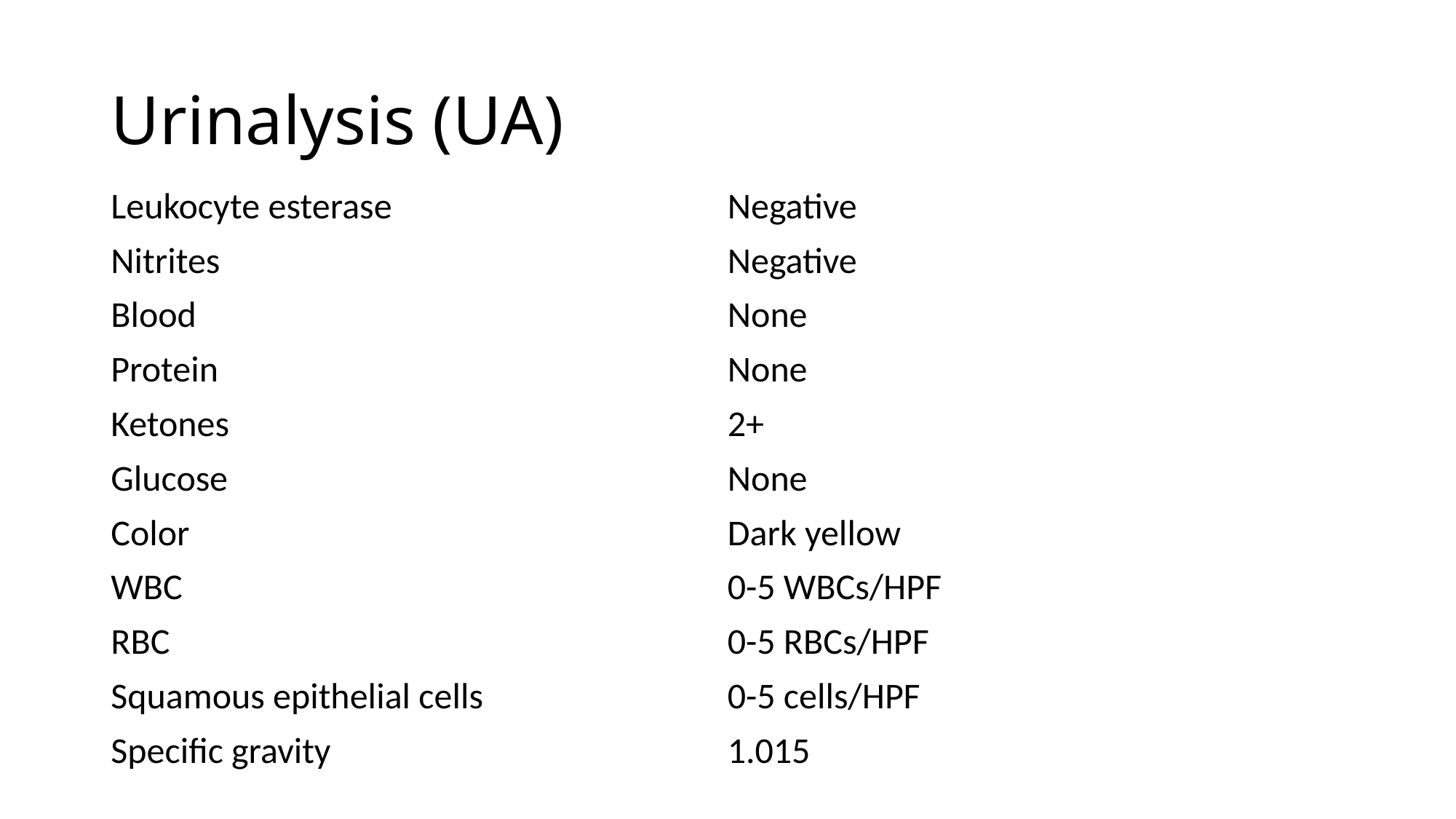

# Urinalysis (UA)
Leukocyte esterase
Nitrites
Blood
Protein
Ketones
Glucose
Color
WBC
RBC
Squamous epithelial cells
Specific gravity
Negative
Negative
None
None
2+
None
Dark yellow
0-5 WBCs/HPF
0-5 RBCs/HPF
0-5 cells/HPF
1.015

## Slide 6
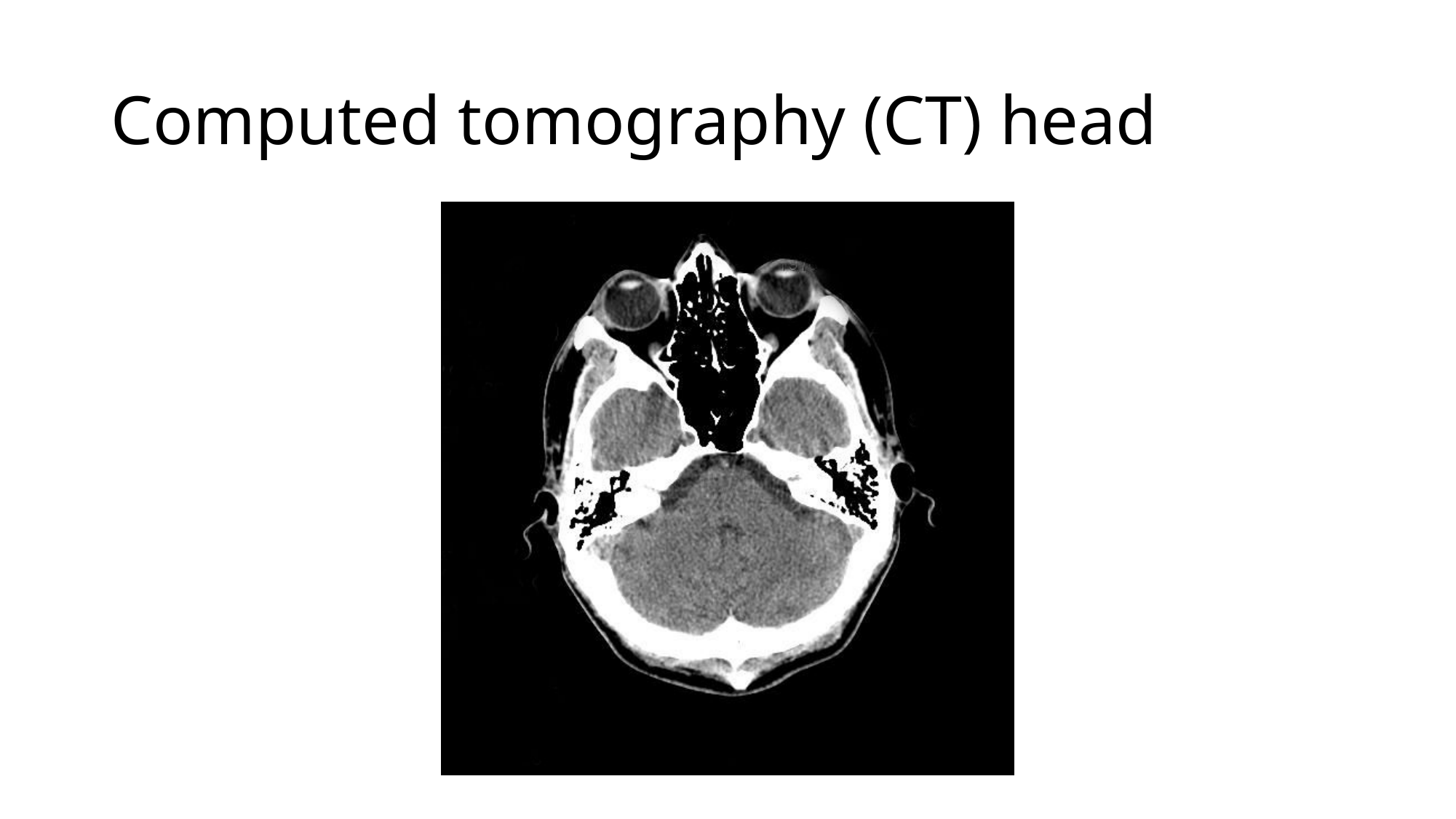

# Computed tomography (CT) head

## Slide 7
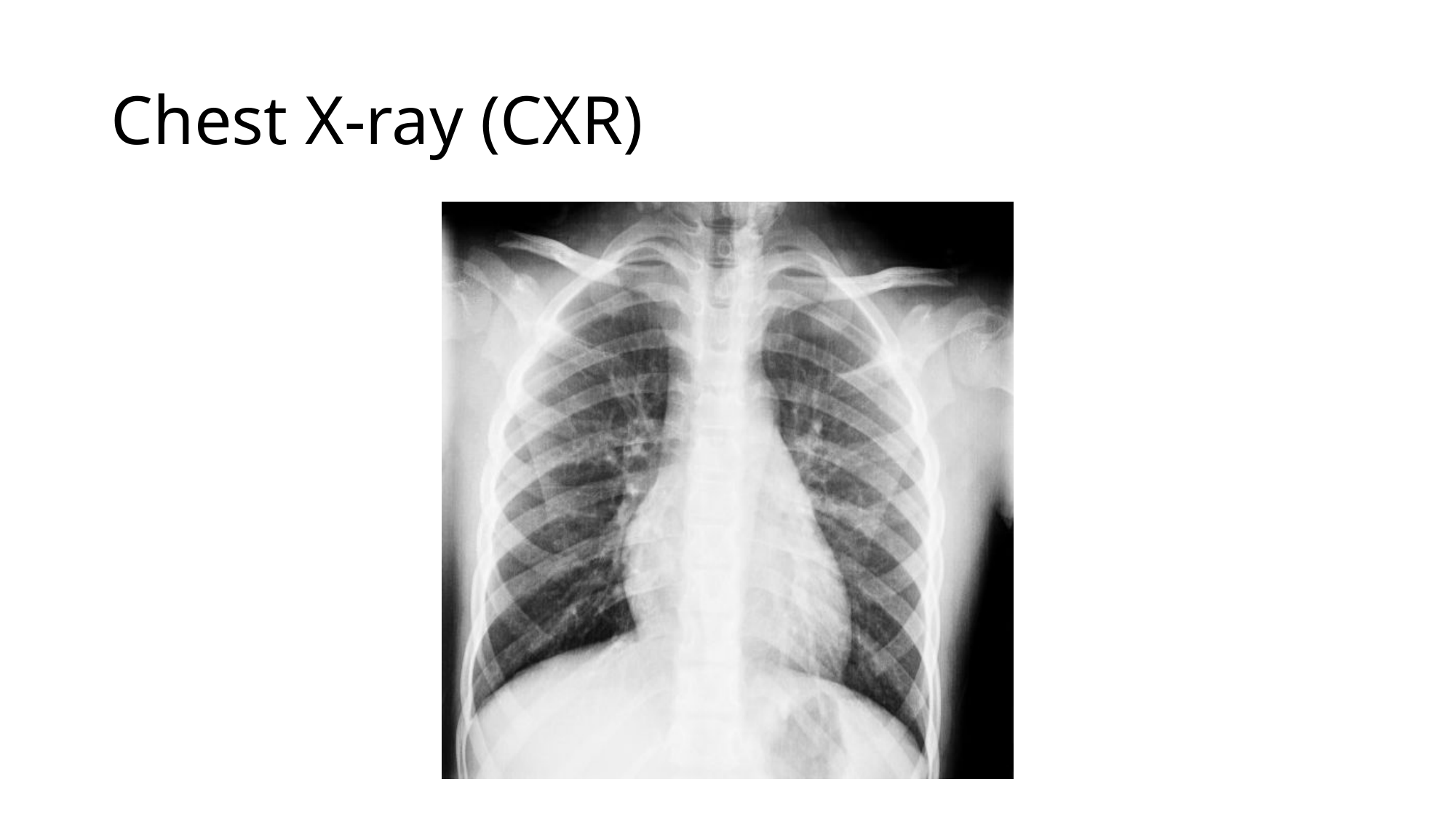

# Chest X-ray (CXR)

## Slide 8
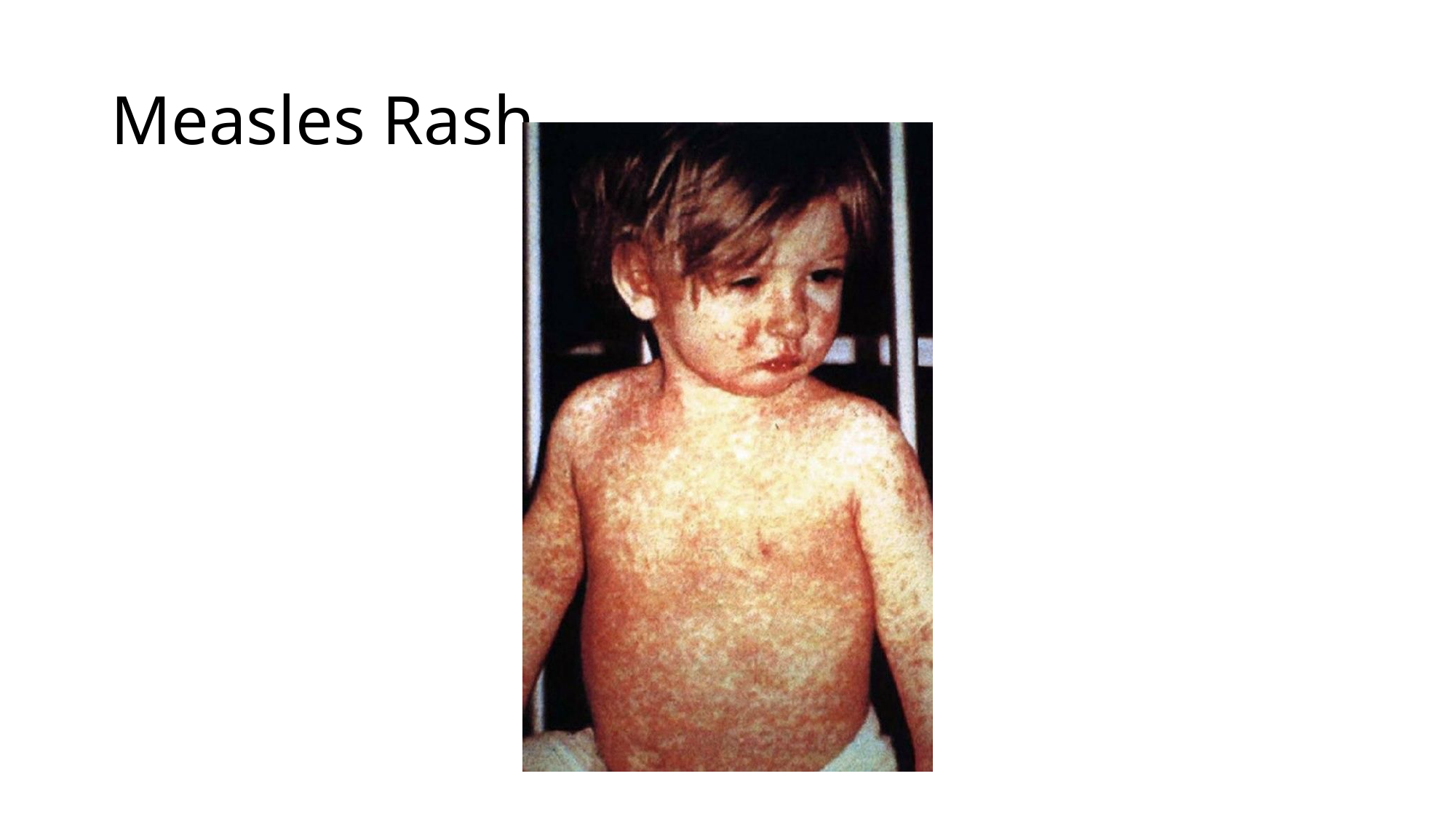

# Measles Rash

## Slide 9
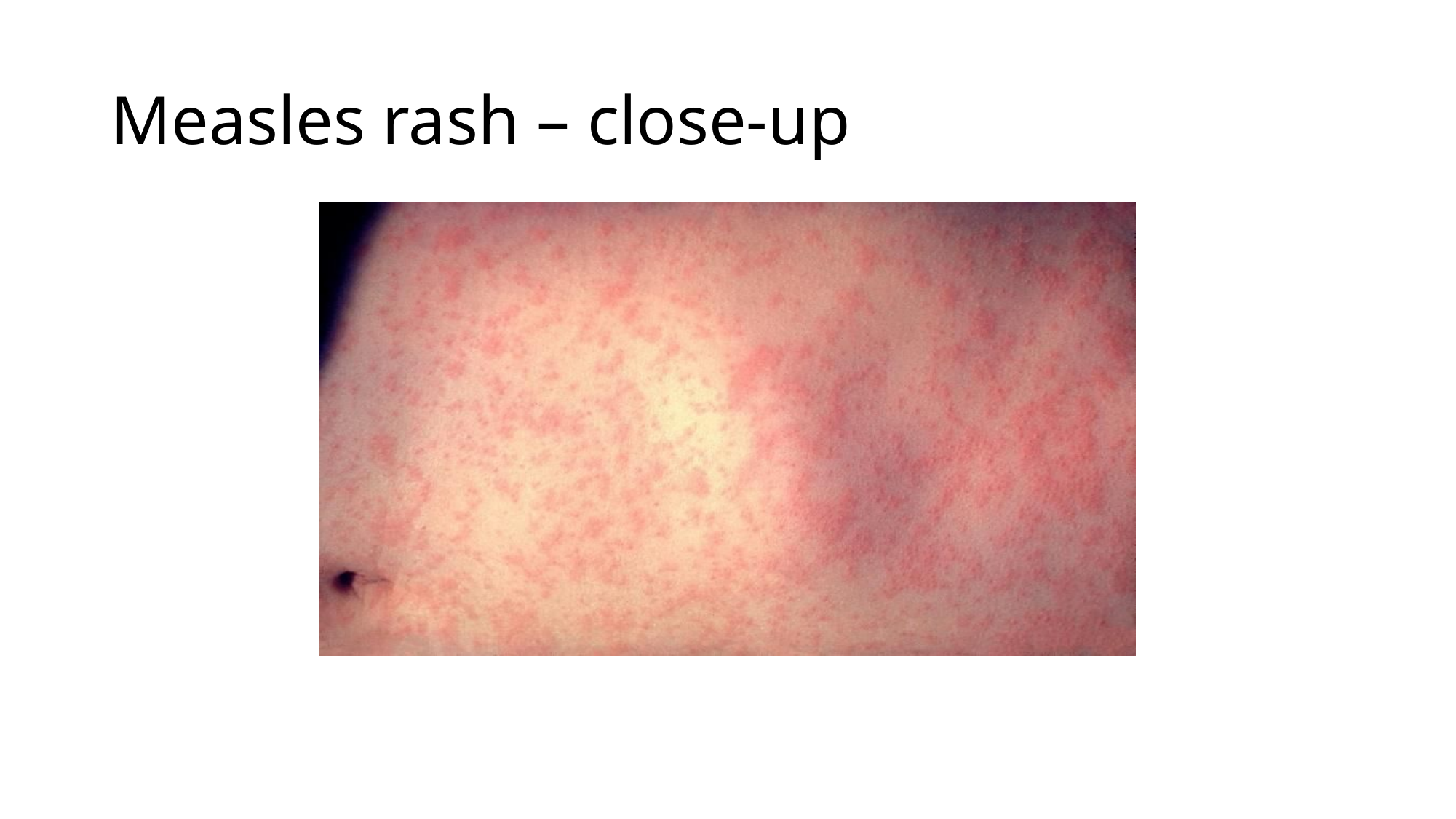

# Measles rash – close-up

## Slide 10
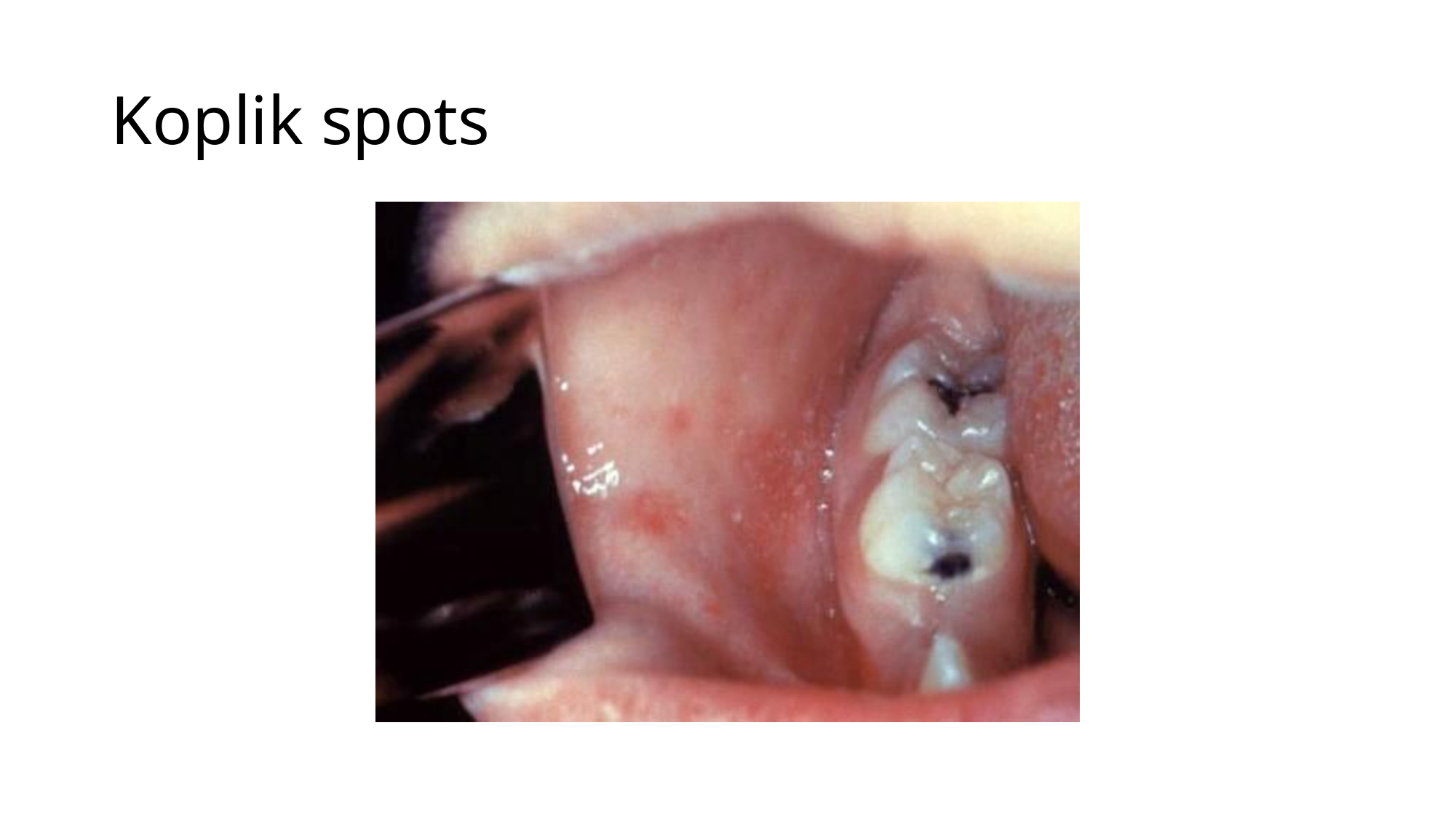

# Koplik spots

## Slide 11
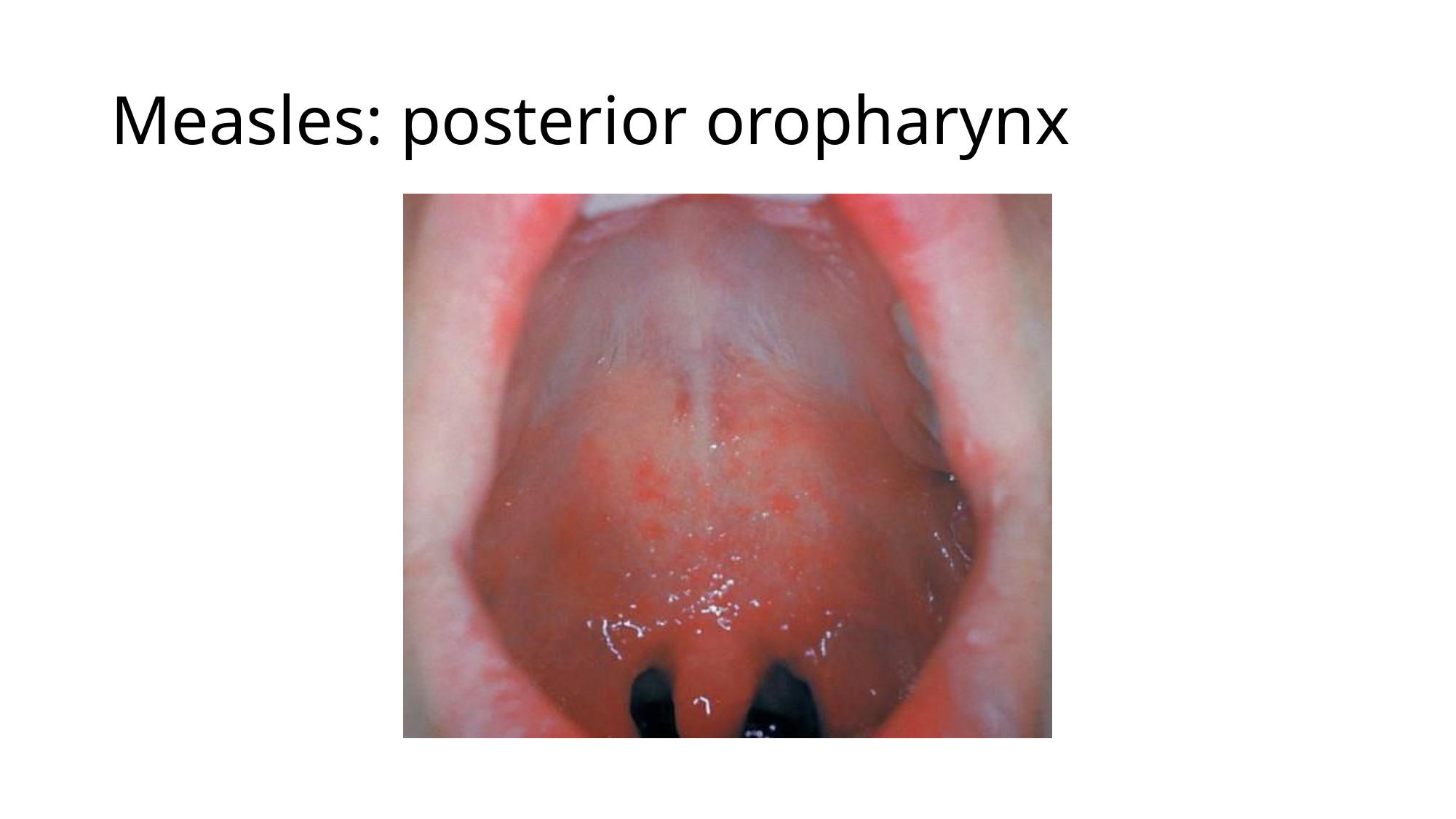

# Measles: posterior oropharynx
